# Characterization of the extent of a large outbreak of Legionnaires’ disease by serological assays

**DOI:** 10.1186/s12879-015-0903-2

**Published:** 2015-03-28

**Authors:** Øystein Simonsen, Elisabeth Wedege, Anita Kanestrøm, Karin Bolstad, Ingeborg S Aaberge, Eivind Ragnhildstveit, Jetmund Ringstad

**Affiliations:** Department of Medicine, Østfold Hospital Trust, Fredrikstad, Norway; Department of Bacteriology and Immunology, Division of Infectious Disease Control, Norwegian Institute of Public Health, Oslo, Norway; Department of Clinical Microbiology, Østfold Hospital Trust, Fredrikstad, Norway

**Keywords:** *Legionella pneumophila*, Outbreak, Serological assays, Community-acquired pneumonia

## Abstract

**Background:**

In May 2005, a long-distance outbreak of Legionnaires’ disease (LD) caused by *Legionella pneumophila* serogroup 1 occurred in south-east Norway. The initial outbreak investigation without serology identified 56 laboratory-confirmed LD cases of whom 10 died. However, 116 patients with community-acquired pneumonia might belong to the outbreak based on epidemiological investigations, but acute laboratory tests other than serology were negative or not performed. To assess the true extent of the outbreak, we evaluated two serological assays in order to reclassify the 116 patients with indeterminate case status.

**Methods:**

Two polyvalent antibody tests, a serogroup 1–6 immunofluorescence assay (IFA) and a serogroup 1–7 enzyme-linked immunosorbent assay (ELISA) were used. They were evaluated with cases defined as culture- or urinary antigen positive LD patients (n = 40) and non-cases defined as confirmed non-LD patients (n = 39) and healthy control subjects (n = 62). The 116 patients, who were negative in culture, polymerase chain reaction and/or urinary antigen tests, were analysed by the same serological assays. Antibodies to the outbreak strain were determined by immunoblotting.

**Results:**

In the evaluation study, the sensitivity and specificity of a ≥4-fold IFA titre change was 38% and 100%, respectively, with corresponding values of 30% and 99% for seroconversion in ELISA. A single high positive IFA titre yielded sensitivity and specificity of 73% and 97%, respectively, with corresponding values of 68% and 96% for a single high immunoglobulin (Ig) G and/or IgM in ELISA.

Based on this evaluation, the following serological testing identified 47 more LD cases, and the outbreak thus comprised 103 cases with a case fatality rate of 10%. About the same proportion (70%) of the urinary antigen positive and negative LD cases had antibodies to the serogroup-specific lipopolysaccharide of the outbreak strain. In addition to the 103 LD cases, *Legionella* infection could not be verified or excluded in 32 patients based on epidemiology and/or lack of microbiological sampling.

**Conclusions:**

The acute-phase tests (culture, polymerase chain reaction, and urinary antigen) identified less than 55% of the 103 patients in this outbreak. Serological testing thus remains an important supplement for diagnosis of LD and for determination of outbreak cases.

## Background

Legionnaires’ disease (LD) has been considered a rare cause of community-acquired pneumonia (CAP) in Norway. The last 10 years, about 6 cases per million inhabitants were reported annually [1] which is lower than the incidence rate of more than 10 per million reported in overall Europe [2]. However, a study in 2005 showed considerable underreporting of the disease in Norway [3]. Insufficient use of diagnostic testing and false negative diagnostic tests may lead to underreporting in passive surveillance systems. A disease incidence of almost 80 per million was estimated in a population-based study [4], and *Legionella* spp. cause between 2% and 16% of CAP cases in prospective studies [5-7], rendering *Legionella* spp. one of the most common pathogens in CAP. *L. pneumophila* serogroup 1 (Lp 1) is responsible for 70% to 90% of all culture positive LD cases [8,9].

Microbiological diagnosis of LD during acute illness is based on culture, polymerase chain reaction (PCR) of respiratory samples, and/or detection of *Legionella* antigen in urine. Isolation of *Legionella* spp. by culture is considered the gold standard for diagnosing LD, but the sensitivity is low. PCR-based methods are rapid and more sensitive than culture [10], but more experience in clinical use is needed [11]. The urinary antigen test (UAT) has become the most performed test in diagnosing LD [8] because of its easy performance and rapidity combined with a reasonable sensitivity ranging from 40% to 94% [12-14]. However, it is only reliable for Lp 1 infections. Serological tests rarely allow a diagnosis during the acute phase and are regarded more useful for epidemiological purposes.

In May 2005, an outbreak of LD caused by Lp 1 (ST15, monoclonal subgroup Benidorm [15]) occurred in south-east Norway [16]. The source was identified as an industrial air scrubber with a long-distance spread of more than 10 km [16]. In addition, the river Glomma has been proposed as a disseminator due to the release of waste water from industrial aeration ponds [17]. The initial outbreak investigation identified 56 patients diagnosed with LD by a positive Lp 1 culture, PCR, and/or UAT [16], but serological testing was not performed. As the aim of that study was mainly to identify the source of the outbreak, probable cases were not included. In the present investigation, sera from the laboratory confirmed LD cases were used to evaluate two commercial serological assays against *L. pneumophila,* which previously had been evaluated in one study only [18]. Based on the sensitivities and specificities obtained in our evaluation, the tests were then employed to measure antibody levels in sera from all CAP patients, who were referred to the local hospital during the outbreak. Our aims were to diagnose additional LD cases and so to determine the extent of the outbreak.

## Methods

### Setting

Østfold Hospital Trust is an acute care hospital that serves the 260,000 inhabitants of Østfold County, including the 120,000 inhabitants of the twin cities Sarpsborg and Fredrikstad. The regional epidemiology of CAP has been constant in May/June during the years 2001 – 2004 with an average of 16 referrals per week (2.3 per day) to the hospital. On 21 May 2005, an LD outbreak was suspected, and the initial outbreak investigation identified 56 cases of LD (53 cases admitted to our hospital and 3 cases to other hospitals in Norway), confirmed by either a positive *Legionella* UAT and/or culture of Lp 1 and/or PCR [16]. In order to identify potentially undiagnosed cases, adult patients referred to our hospital with radiographically confirmed CAP during a five-week period from 9 May to 12 June 2005 were invited to a serological study. The period chosen was based on the previous report of this outbreak [16]. In addition, healthy controls, recruited from the hospital staff, were invited in late May for evaluation of the serological assays. The study was approved by the Regional Committee for Medical and Health Research Ethics (REC) South East, P.O BOX 1130, Blindern, NO 0318 Oslo, Norway. Written informed consent for participation in the study was obtained from all participants.

### Microbiological investigation

Blood cultures were drawn from CAP patients referred to the emergency department during the five-week period, and sputum cultures were sampled from patients with expectoration. Isolated bacterial species were classified as an aetiological pathogen if predominant growth of a typical respiratory tract pathogen was observed. Lung tissue specimens from deceased patients and sputum were also analysed by a commercial PCR test (Onar Lp-QP; Minerva Biolabs GmbH, Germany) for detection of *Legionella* spp. and cultured for *Legionella* (plated both directly and after an acid decontamination step onto selective and non-selective buffered charcoal-yeast extract agar). Isolates were serogrouped with *Legionella* Latex Test (Oxoid, Basingstoke, UK).

Urine samples from patients and healthy control subjects were analysed by the Now *Legionella* Urinary Antigen Test and the Now *Streptococcus pneumoniae* Urinary Antigen Test (Binax, Portland, Maine) for qualitative detection of soluble *Legionella* and *S. pneumoniae* antigen, respectively.

Sera from CAP patients were collected during the acute phase (0 – 15 days after symptom onset) if available, 4 – 6 weeks, and 3 months after hospital admission. Sera from healthy employees were collected in late May and after approximately 1 and 3 months. All sera were stored at −20°C. Two commercial polyvalent serological *L. pneumophila* assays were used for evaluation of the methods and the outbreak investigation: a serogroup 1–6 immunoglobulin (Ig) G/IgM/IgA immunofluorescence assay (IFA) (Meridian Bioscience Europe, Milan, Italy) and a serogroup 1–7 enzyme-linked immunosorbent assay (ELISA) (Serion ELISA classic, Institut Virion/Serion GmbH, Würzburg, Germany) with separate levels of IgG and IgM antibodies measured in an ELISA robot (DSX Automated System, Dynex Technologies, Inc, Virginia). The coefficients of variation for four standard samples per plate in this study were 7.6% for IgG (range 4.1 to 10.0) and 6.6% for IgM (range 5.8 to 7.4) compared with the maximal interserial coefficient of variation of 16% given by the manufacturer [19]. Both tests were performed according to the manufacturers’ instructions and analysed without knowledge of Lp 1 infection status.

### Evaluation of the serological assays

The reference standard for the serological evaluation was based on the EU case definition [20], but excluding serology as microbiological evidence of infection. Hence, patients with radiographically confirmed CAP admitted during the defined five-week period and with either isolation of *L. pneumophila* from a respiratory sample and/or a positive *L. pneumophila* UAT were defined as cases. Patients with confirmed non-LD pneumonia and healthy control subjects were considered to be non-cases. The definition of confirmed non-LD pneumonia was radiographically documented CAP and a negative *Legionella* UAT and culture with either a) proven aetiology other than *Legionella*, b) date of symptom onset before 10 May or after 27 May 2005 (based on the symptom onset period of the initial 56 cases), and/or c) residency outside and not visiting the outbreak area which was defined by the plume model from the aerosol dispersion investigation [16]. Only LD cases and healthy controls with paired serum samples were included in the evaluation study.

The assays were evaluated from the antibody responses corresponding to the EU laboratory criteria for confirmed and probable cases in addition to an alternative ELISA-ratio method:

1) Serologically confirmed LD was defined as a ≥ 4-fold change in IFA titres in paired sera or seroconversion in ELISA to a positive IgG (≥50 U/ml) or IgM (≥120 U/ml), which include the borderline ranges given by the manufacturer [19].

2) Serologically probable LD was defined as a single high or high standing antibody level with an IFA titre ≥128, as given by the manufacturer, and/or ELISA IgG ≥50 U/ml and/or IgM ≥120 U/ml.

3) An alternative definition of probable LD was based on a relative change of IgG and/or IgM antibody levels in paired serum samples in ELISA and calculated for all paired serum samples including antibody levels below the manufacturer’s cut-off. To exclude clinically irrelevant changes in the low antibody ranges, only sera with IgG or IgM levels above the 75th percentile of the non-cases (IgG 16 U/ml and IgM 26 U/ml, respectively), were considered for this calculation. In paired sera with antibody levels below these values, the ratio was set to 1.0, indicating no change.

### Outbreak investigation

Sera from patients with CAP, who lived within or visited the outbreak area, but were negative in *Legionella* culture, PCR, and/or UAT, were analyzed with the serological assays. From their antibody responses, patients with positive serology were defined as confirmed or probable LD cases and included in the outbreak.

### Classification of pneumonia severity

Pneumonia severity was classified by CRB-65 [21], a score derived by four criteria of severity obtained on admission: **c**onfusion, **r**espiratory rate (>30 per minute), low **b**lood pressure (diastolic pressure ≤60 mmHg or systolic pressure ≤90 mmHg), and age ≥65 years. A CRB-65 score ≥2 was considered to be moderate to severe pneumonia.

### Immunoblotting

To study if the antibody responses, determined in ELISA and IFA with the polyvalent antigens, were directed to the serogroup-specific lipopolysaccharide (LPS) antigen of the Lp 1 outbreak strain [22], immunoblotting with whole-cell suspensions of this strain was performed as described previously [23] with detection of IgG and IgM binding on separate strips. From each patient, the serum sample (diluted 1:200) with the highest IgG or IgM antibody level in ELISA was used. Blotting was also performed with proteinase K (Qiagen Gmbh, Hilden, Germany) treated cells [24] from the outbreak strain and from an Lp 1 isolate of subgroup France/Allentown to study LPS cross-reactive antibodies. The LPS patterns of the strains were obtained by silver–staining of sodium dodecyl sulphate polyacrylamide gels [25] as well as by incubation of strips with an Lp 1 monoclonal antibody from the Dresden panel [26]. Antibody binding to the corresponding LPS region on the strips was recorded visually as strong, weak, or no response.

### Statistical methods

Statistical analyses were performed with SPSS version 16.0. Comparisons between groups were performed using the χ^2^ test or independent t-test when appropriate. All tests were two-sided, and the significance level was set at 0.05. Diagnostic sensitivity and specificity were calculated for each test using OpenEpi [27]. Correlation between the two serological assays was calculated by the Spearman rank order test.

## Results

### Evaluation of the serological assays

**Figure 1:**
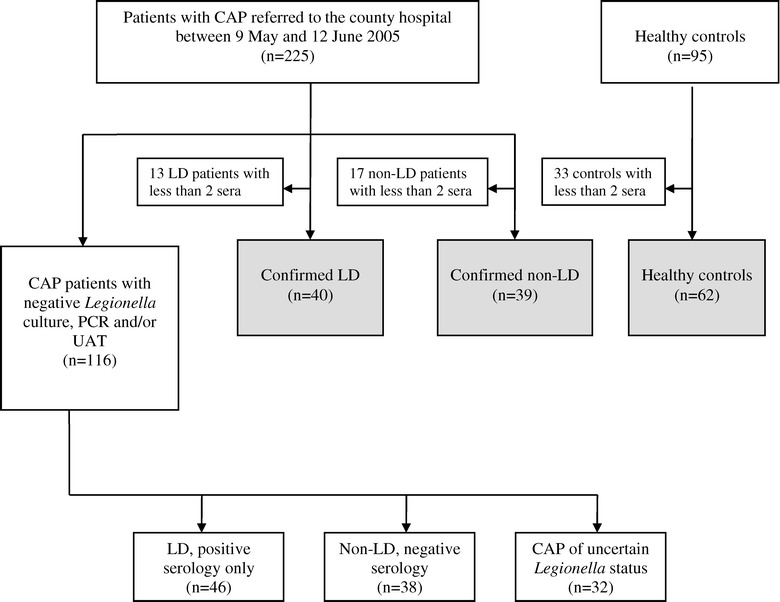
**Selection of eligible patients for the serological evaluation study.** Only individuals with ≥2 paired sera were selected for this study (grey boxes). Non-LD patients and healthy controls formed the non-cases. Also shown are the serological results for the 116 CAP patients with negative *Legionella* culture, PCR, and/or UAT. Four LD cases (three diagnosed with UAT during the acute phase and one with positive serology and negative UAT) were admitted to other hospitals in Norway and are not included in the figure. CAP: community-acquired pneumonia. UAT: *Legionella* urinary antigen test. LD: Legionnaires’ disease (confirmed by *Legionella* culture, PCR and/or UAT). Non-LD: CAP of non-*Legionella* aetiology.

A total of 225 adult patients with CAP of any cause was referred to our hospital during the defined five-week period. Before serological testing was performed, LD and non-LD pneumonia were confirmed in 53 and 56 patients, respectively, leaving 116 patients with unknown aetiology (Figure 1). Non-LD was confirmed by the finding of another respiratory pathogen (16 patients), date of illness onset before 10 May or after 27 May (35 patients), and residency outside and not visiting the outbreak area (5 patients). The patients included for the serological assay evaluation comprised 40 LD cases (median age 68 years, range 35 – 94) and 101 non-cases (39 non-LD patients, median age 68 years, range 18 – 88, and 62 healthy controls, median age 45 years, range 24 – 62) from whom two or more sera were collected (Figure 1).

Sensitivities and specificities of the two serological assays for the different LD case definitions are shown in Table 1. The IFA and ELISA results were roughly equal for both the confirmed and probable LD case definitions, and combination of both assays resulted in somewhat better sensitivity without affecting the specificity. The two serological assays also demonstrated significant correlations between the IFA titres and the sum of IgG and IgM levels in ELISA with a Spearman rank correlation coefficient of 0.79 (*P* < 0.001).

**Table 1:** **Diagnostic sensitivity and specificity of IFA and ELISA tests for different Legionnaires’ disease case definitions**

**Case definition**	**Serological response**	**Sensitivity (95% CI) n = 40**	**Specificity (95% CI) n = 101**
Serologically confirmed LD	≥4-fold titre change in IFA	38 (24 – 53)	100 (96 – 100)
	IgM and/or IgG seroconversion in ELISA	30 (18 – 45)	99 (95 – 100)
	Combination of IFA and ELISA	53 (38 – 67)	99 (95 – 100)
Serologically probable LD (single high or high standing antibody level)	IFA-titre ≥128	73 (57 – 84)	97 (92 – 99)
	ELISA IgM ≥120 U/ml and/or IgG ≥50 U/ml	68 (52 – 80)	96 (90 – 98)
	Combination of IFA and ELISA	78 (63 – 88)	96 (90 – 98)
Serologically probable LD (≥1.5 ratio in ELISA)	IgM-ratio ≥1.5	70 (55 – 82)	100 (96 – 100)
	IgG-ratio ≥1.5	63 (47 – 76)	98 (93 – 99)
	IgM-ratio ≥1.5 and/or IgG-ratio ≥1.5	83 (68 – 91)	98 (93 – 99)

LD: Legionnaires’ disease. Data on sensitivity and specificity are in percent and based on 40 patients with confirmed LD from culture, PCR, and/or UAT and 101 non-cases (39 non-LD pneumonia patients and 62 healthy controls).

In the alternative ELISA evaluation, the median IgG- and IgM ratios were 1.7 (interquartile range (IQR) 1.1 – 2.7) and 3.1 (IQR 1.1 – 6.2), respectively, for the LD cases with corresponding values of 1.04 (IQR 1.00 – 1.14) and 1.09 (IQR 1.00 – 1.24) for the non-cases. The highest combined sensitivity and specificity were found for a ratio of >1.5 in paired serum samples for both IgG and IgM (Table 1).

We found no statistically significant association between pneumonia severity score and sensitivity of the serological assays (*P >* 0.27) for any of the case definitions.

### Outbreak investigation

During the initial outbreak investigation [16], 116 of the 225 patients referred to our hospital could not be classified definitely into LD or non-LD based on *Legionella* culture, PCR and/or UAT and epidemiological criteria (Figure 1). Following the evaluation study, these patients were studied by the serological assays using the three case-definitions as the specificity of more than 96% (Table 1) in an epidemic setting was considered acceptable.

Table 2 shows the results of all utilized tests. Fifty-six cases were previously diagnosed with LD by the acute phase tests [16], of which UAT was the most important. PCR and culture of sputum and lung tissue were positive in eight and ten cases, respectively, but the additive contribution of these tests was limited to four cases. However, isolation of Lp 1 was essential for the investigation of the source [16].

**Table 2:** **Diagnostic tests for Legionnaires’ disease in 103 outbreak patients**

**Case definition**	**Laboratory test**	**No. positive/no. tested (%)**	**Additional (cumulative) no. of LD-cases**	**Comments**
Confirmed LD (direct demonstration of Lp 1 infection)	Positive Lp 1 UAT*	52/99 (53)	52	
	Culture of Lp 1 (sputum)*	4/30 (13)	1 (53)	Pos. UAT : 3
				Neg UAT: 1
	Culture of Lp 1 (lung tissue)*	6/7 (86)	3 (56)	Pos UAT: 3
				Neg UAT: 2
				UAT not done: 1
	Positive PCR	8/25 (32)	0 (56)	Sputum: 5/22
				Lung tissue: 3/3
Confirmed LD** (serology)	≥4-fold titre change in IFA	40/84 (48)	25 (81)	13 patients had ≥4-fold titre change in IFA, but no seroconversion in ELISA.
	IgM/IgG seroconversion in ELISA	30/84 (36)	6 (87)	
Probable LD (single high or high standing antibody level)	IFA-titre ≥128	68/92 (74)	9 (96)	
	ELISA IgM ≥120 U/ml and/or IgG ≥50 U/ml	59/92 (64)	1 (97)	
Probable LD (ELISA ratio ≥1.5)	ELISA IgM ratio ≥1.5 and/or IgG ratio ≥1.5	75/84 (89)	6 (103)	

LD: Legionnaires’ disease, Lp 1: *L. pneumophila* serogroup 1, UAT: urinary antigen test. The proportion of serogroup 1 specificity to the outbreak strain on immunoblots among the serologically confirmed and probable LD cases was similar (70%) to that of the culture, PCR, and/or UAT-confirmed LD cases.

*Historical data [16]. Three of these patients were admitted to other hospitals in Norway and thus not shown in Figure 1.

**One of these cases was admitted to another hospital and thus not shown in Figure 1.

Serologically confirmed or probable LD was found in 47 patients (Table 2). Thirty-one of the cases had confirmed LD demonstrated by a ≥4-fold titre change in IFA or seroconversion in ELISA. One of these, a local resident from the outbreak area, was admitted to another hospital and therefore not included in Figure 1. Ten cases had probable LD shown by a single high or high standing antibody titres. The alternative use of ELISA IgG/IgM-ratios, added only six cases to the total number. Thus, our data indicated that the outbreak comprised at least 103 cases.

LD was ruled out in 38 patients based on negative serology, whereas *Legionella* infection could not be verified or excluded in the remaining 32 CAP patients (Figure 1). Twelve of these patients refused participation, seven had cognitive failure, ten died without microbiological sampling, and three had positive serology consistent with probable LD but died before *Legionella* exposure could be clarified, and neither UAT nor autopsy was performed.

The mean age for the 103 LD cases was 67 years (range 35 – 94), and there were 64 males and 39 females. Ten of the cases, with a median age of 80 years (range 69 – 94), died during hospitalisation (case fatality rate 10%). The demographic and serological results in Table 3 showed few significant differences between the two LD patient groups diagnosed by culture, PCR, and/or UAT and serology, respectively. The latter group had less severe pneumonia, no deaths, and less frequent intensive care unit (ICU) admission than LD cases diagnosed in the acute phase. No statistically significant differences in antibody levels were observed between the two groups except for a higher IgM level at 3 months among cases with a positive culture/PCR/UAT.

**Table 3:** **Demographic and serological data among LD patients diagnosed by acute phase tests and serology**

	**Positive culture/PCR/UAT (n = 56)**	**Positive serology only (n = 47)**	***P-*** **value**
Age, mean years ± SD	69 ± 14	65 ± 12	0.15
Male gender	34 (61)	30 (64)	0.75
Chronic respiratory disease	11 (20)	11 (23)	0.64
Active smoker	22 (39)	17 (36)	0.69
Previously healthy	20 (36)	18 (38)	0.79
Date of symptom onset	16. May	16. May	
(interquartile range [IQR])	(14.–19. May)	(14.–19. May)	0.90
Inpatient treatment	55 (98)	45 (96)	0.45
C-reactive protein (mg/L) ± SD	296 ± 116	237 ± 89	0.15
ICU-admission	19 (34)	2 (4)	<0.001
CRB-65 score ≥2	33 (59)	16 (34)	0.012
Infiltrate > one lobe	13 (23)	9 (19)	0.83
Mortality	10 (18)	0 (0)	0.002
IFA-titre, acute phase	64 (0 – 256)	32 (0 – 64)	0.16
IFA-titre, after 1 month	256 (64 – 1024)	128 (128 – 256)	0.11
IFA titre, after 3 months	128 (32 – 1024)	64 (32 – 128)	0.15
ELISA IgG, acute phase	41 (10 – 115)	20 (9 – 52)	0.25
ELISA IgG, after 1 month	53 (27 – 188)	66 (30 – 193)	0.96
ELISA IgG, after 3 months	60 (17 – 312)	33 (12 – 131)	0.20
ELISA IgM, acute phase	24 (4 – 93)	8 (3 – 150)	0.15
ELISA IgM, after 1 month	47 (22 – 195)	29 (16 – 87)	0.11
ELISA IgM, after 3 months	21 (10 – 76)	11 (6 – 30)	0.04

Demographic data are no. (%) of patients unless otherwise indicated. Serological results are given in median (interquartile range) titre for IFA and U/ml for ELISA IgG and IgM.

ICU: Intensive care unit. CRB-65 score ≥2 indicates moderate to severe pneumonia.

**Figure 2:**
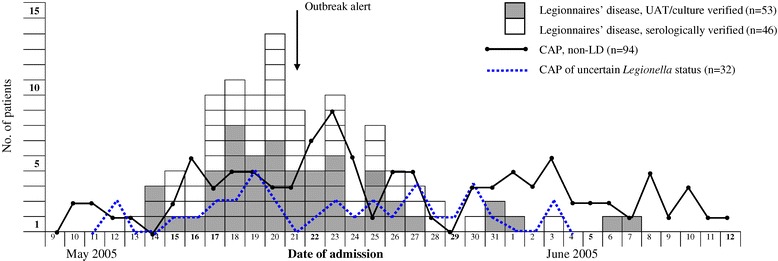
**Cases of Legionnaires’ disease and community-acquired pneumonia (CAP) by date of admission to the hospital.**

Compared with all 94 non-LD patients (56 patients with confirmed non-LD and 38 patients with negative serology, Figure 1), LD cases had more severe pneumonia (*P* = 0.048) and a higher proportion of ICU admission (*P* = 0.019) than non-LD patients (Table 4). However, chronic respiratory disease was more frequent in the non-LD group (*P* = 0.002). The group of 32 CAP patients with uncertain *Legionella* status had a high mean age and mortality, but statistical comparisons of this group with the two others are less reliable due to its heterogeneity as described above. Figure 2 demonstrates the hospitalisation date for patients with LD diagnosed by culture/PCR/UAT and serology, respectively, non-LD, and CAP of unknown aetiology during the defined five-week period. The number of admissions for LD cases mirrored the outbreak epidemic curve [16]. The non-LD admission curve, which was expected to fluctuate around a mean of 2.3 per day based on hospitalization rates in 2001 – 2004, also peaked, but two days after the outbreak alert. This curve indicated an over-referral of approximately 15 non-LD patients probably caused by the media attention following the outbreak. The 32 patients with CAP of unknown aetiology were scattered throughout the period with a peak two days before the outbreak alert.

**Table 4:** **Demography of different patient groups referred to the hospital during the outbreak**

	**Legionnaires’ disease (n = 103)**	**CAP, non-** ***Legionella*** **(n =94)**	**CAP of uncertain** ***Legionella*** **status (n = 32)**
Age, mean years ± SD	67 ± 13	65 ± 17	75 ± 16
Male gender	64 (62)	50 (53)	15 (47)
Active smoker	39 (38)	28 (30)	6 (19)
Chronic respiratory disease^1^	22 (21)	39 (41)	12 (38)
Diabetes	16 (16)	12 (13)	3 (9)
Previously healthy	38 (37)	26 (28)	6 (19)
Inpatient treatment	100 (97)	85 (90)	29 (91)
ICU-admission^2^	21 (20)	8 (9)	4 (13)
CRB-65 score ≥2^3^	48 (47)	28 (30)	15 (47)
Infiltrate > one lobe	23 (22)	12 (13)	3 (9)
Mortality	10 (10)	6 (6)	6 (19)

^1^ *P* = 0.002.

^2^ *P* = 0.019.

^3^ *P* = 0.048.

Patients with Legionnaires’ disease, community-acquired pneumonia (CAP) of non-LD aetiology and CAP of uncertain *Legionella* status referred to hospital from 9 May to 12 June are shown. Data are no. (%) of patients unless otherwise indicated. *P*-values in footnotes indicate the comparison between Legionnaires’ disease and CAP of non-*Legionella* aetiology.

### Lp 1 specificity of LD case sera

**Figure 3:**
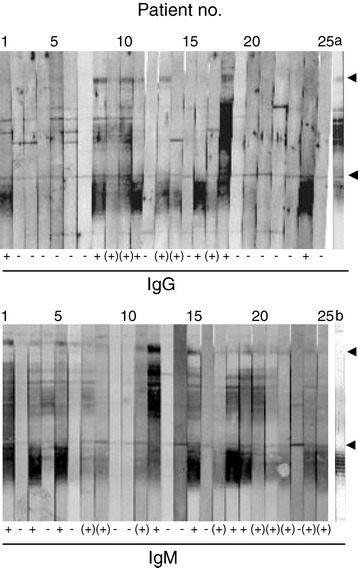
**IgG and IgM binding to the outbreak strain with sera from UAT negative LD cases.** The immunoblots show IgG and IgM antibody binding, respectively, with sera from 25 UAT-negative cases to whole cells of the *L. pneumophila* serogroup 1 outbreak strain. Individual cases are identified by numbers above the nitrocellulose strips, and the upper and lower arrows to the right show the positions of proteins of molecular masses of approx. 80 kDa and 25 kDa, respectively. Strip a: binding of a monoclonal antibody to serogroup 1 *L. pneumophila* (Lp 1 from the Dresden panel [26]); strip b: IgM antibody reactions of serum from case no. 25 from another experiment with proteinase K treated cells, showing the ladder-like LPS antibody responses. IgG and IgM binding intensities of each serum to LPS are rated below the strips as + (strong), (+) (weak), and – none. Each 12% acrylamide gel was loaded with whole cells from the outbreak strain, corresponding to 2 μg protein/strip, and the strips were incubated with 1:200 serum dilutions. UAT: urine antigen test.

Immunoblotting was performed to test whether the antibody responses observed with the polyvalent ELISA and IFA were directed to the serogroup-specific LPS of the Lp 1 outbreak strain. Sera from 44 culture/UAT-positive and 45 UAT-negative/serology positive LD cases were available for immunoblotting. Figure 3 shows the IgG and IgM responses with the outbreak strain for 25 randomly chosen UAT-negative cases. Antibody binding to the LPS region below 25 kDa dominated in addition to individual antibody reactions in the 25 to 80 kDa molecular mass range. For IgM in particular, the binding intensities to the LPS region below 25 kDa corresponded with intensities of a smear in the higher molecular weight range. Among all 45 UAT-negative cases, 30 (67%) had IgG and/or IgM antibodies that showed strong (16 cases) or weak binding (14 cases) to LPS on the blots. A similar proportion of the UAT-positive cases/(30/44; 68%) also showed corresponding LPS bands (19 and 11 cases with strong and weak bands, respectively), whereas no such reactions were observed with sera from the 39 confirmed non-LD patients (data not shown). LPS band intensities generally corresponded to the IgG or IgM antibody levels in ELISA. Proteinase K treatment of the outbreak strain gave similar LPS responses as the untreated cells, but the distinct high molecular weight bands were absent and the ladder-like LPS pattern below 25 kDa became more distinct after proteolysis (Figure 3; strip b). In comparison with these LPS responses, 15 (33%) and 26 (59%) of the sera from the UAT-negative and UAT-positive cases, respectively, demonstrated strong or weak LPS bands with a proteolytically treated serogroup 1 France/Allentown isolate (data not shown). Thus, more UAT-positive cases seemed to have cross-reactive antibodies to this strain than the UAT-negative cases.

## Discussion

This Legionella outbreak demonstrated the challenges of the microbiological diagnosis of LD: 1) the acute phase tests (culture, PCR, and UAT) underestimated the number of cases; 2) the traditional serological criterion of a ≥4-fold titre increase yielded poor sensitivity in this outbreak population; and 3) even when all diagnostic tests were employed, there was still an unexplained excess of hospitalized CAP patients in whom *Legionella* infection could not be verified or excluded indicating shortcomings of the microbiological tools. Previously, the acute phase tests diagnosed 56 LD outbreak cases [16]. Our study identified an additional 47 cases with the serological assays, thus the outbreak comprised a total of 103 LD cases, of whom 87 were confirmed and 16 were probable cases. Another group of 32 patients with CAP of high mortality may also belong to the outbreak.

UAT has emerged as the most common test for laboratory diagnosis of LD [2] because of its simplicity and high specificity. Although it was useful during the acute phase, UAT was positive in only 52 of the 99 cases (53%) who were tested (Table 2). This number corresponded to those from two other outbreaks [28,29] and a recent methodological study [30]. However, a meta-analysis [14] reported a sensitivity of 74%, and in infections caused by Lp 1 strains with the virulence-associated epitope, recognized by the 3/1 monoclonal antibody [26] including the outbreak strain [15], the sensitivity was more than 90% [13]. As UAT has lower sensitivity in mild LD [12], the sensitivity discrepancy is probably due to the broader clinical spectrum including less severe CAP in the outbreak population. Besides UAT, only four more cases were identified by culture of sputum and lung tissue and none by PCR, but fewer samples were analysed by these two assays (Table 2).

Evaluation of the serological assays (Table 1) demonstrated lower sensitivity than Yzerman *et al.* [18] reported with LD patients from the large Dutch outbreak with the same assays, especially for the ≥4-fold IFA-titre rise (38% vs. 61%) and ELISA seroconversion (30% vs. 64%). However, the results were more similar for the high standing titre definition (73% vs. 86% in IFA and 68% vs. 75% in ELISA). Early sampling may lead to higher sensitivity; in our study, the mean time from disease onset to sampling of the acute-phase sera was 11 days (range 5 – 15) compared with 8 days (range 0 – 15) in the Dutch study [18]. This suggested that seroconversion may already have occurred, especially for IgM antibodies [31]. The use of paired sera from LD cases in the evaluation study (Table 1) further indicated the presence of exclusion bias as the most severely ill LD cases died before convalescent sera could be sampled. Thus, the low mortality among the LD cases in our evaluation study (0% vs. 9% in the Dutch study), indicating differences in illness severity, and the different Lp 1 strains in the two outbreaks [18] may present alternative explanations for the divergent results. Our study did not demonstrate any association between the sensitivity of the serological tests and pneumonia severity, but the statistical power was low.

The alternative ELISA evaluation, based on the ratios in paired samples of IgG and IgM ≥ 1.5, respectively, showed the highest combined sensitivity and specificity of the three evaluated case-definitions (Table 1). The higher sensitivity was obtained because several cases with high ratios were negative according to the manufacturer’s cut-off. A similar method has been validated in ELISA for infections caused by *Mycoplasma pneumonia* and *Chlamydophila pneumonia* [32,33], but to our knowledge, this method, which detected six additional LD cases (Table 2), has not previously been applied for *Legionella* testing. As about 20-30% of LD patients do not develop significantly increased antibody levels even after prolonged observation [34], the advantage of this method is to ignore an absolute cut-off level which must be set high in order to produce a low level of false positives. The challenge remains to find a reasonable cut-off to avoid clinically irrelevant changes in the low antibody range, and the specificity will probably suffer in a non-outbreak study.

Selection of non-cases for evaluation of the specificity was not optimal. Ideally, this group should consist of non-LD CAP patients only, but misclassification was difficult to avoid because of the epidemiological situation and the low negative predictive value of a negative UAT. We therefore included healthy controls to increase the power of the specificity analysis. Although this group was also exposed to the *Legionella* outbreak, as demonstrated by the slightly higher antibody levels among healthy blood donors in the outbreak county compared with donors in a non-exposed county [23], it differed from the CAP population in both age and past medical history which might have affected the results. There was no seroconversion in the healthy control group, and the antibody levels (median IgG 9 U/ml, range 1 – 111 U/ml, and median IgM 14 U/ml, range 2 – 82 U/ml) were comparable to those demonstrated one year after the outbreak in healthy blood donors from the same county [23].

As the polyvalent antigens in the ELISA and IFA may affect the case definitions, immunoblotting with the Lp 1 outbreak strain was performed to investigate if the antibody responses were directed to the serogroup-specific LPS antigen of this strain. These experiments showed that two-thirds of both the UAT-positive and the UAT-negative LD cases had IgG and/or IgM antibodies that reacted with the LPS. About the same proportion of the UAT-positive cases, but only 30% of the UAT-negative cases showed cross-reactive antibodies with LPS of a subgroup France/Allentown Lp 1 strain that also carry the virulence-associated monoclonal 3/1 epitope [26]. It is less likely that this cross-reaction is caused by infection of France/Allentown strains as these are rarely seen in Norway after 2001 [15]. Some cross-reactions may probably occur as various subgroups of Lp 1 strains show the same ladder-like profiles in silver-stained gels [35]. A rabbit serum to one Lp1 subgroup was found to cross-react with the other subgroups on immunoblots, but a corresponding reaction was only observed with a few of the 14 serogroups of *L. pneumophila* [35,36]. In Norway, the incidence of LD is low, and only a small number of seroresponders (2.3%) among healthy blood donors was observed in the outbreak county [23]. Together with the same antibody levels (Table 3) in the UAT positive and negative cases, the same LPS-specific antibody responses, the same time period, and home addresses, our findings suggested that the UAT-negative LD cases were most likely infected with the outbreak strain and not with other *L. pneumophila* strains.

This LD outbreak probably included more than the 103 verified cases. The average number of patients referred to our hospital with CAP during the same five-week period in the preceding four years was 80 (range 72–86). Comparison of this number with the 225 CAP patients admitted during the outbreak in 2005 (Figure 1), indicated an excess of 145 patients and possible LD cases. This observation may be supported by another outbreak study which suggested that UAT and culture detected less than 40% of the LD patients, when the unexplained excess of CAP patients with possible LD was taken into consideration [29]. Based on a similar assumption for this outbreak (56 cases originally detected = 40% of the outbreak), we might expect to find about 140 patients with LD. A major limitation of these calculations was the uncertainty of increased referral because of the outbreak alert. However, no more than 15 patients could be attributed to this effect as indicated by Figure 2. Compared with the previous four years, the proportion of outpatient treatment was approximately the same indicating comparable referral and admission strategies. Furthermore, in 32 hospitalized CAP patients with high mortality, *Legionella* infection could not be ruled out based on epidemiological or laboratory criteria. A considerable proportion of these was admitted during the main bulk of referrals immediately before the outbreak was recognized (Figure 2). This finding suggested that the incidence is underestimated even in prospective studies. Underdiagnosis of non-severe LD might not seem important as the mortality is low. However, recovery from LD is poorly studied, but might include reduced pulmonary function [37,38], fatigue, and even posttraumatic stress disorder [39].

## Conclusions

Our study demonstrated that serological testing is a valuable tool to determine the total number of patients during an LD outbreak investigation. The serological assays detected 47 more cases than the 56 cases previously diagnosed by culture, PCR, and/or UAT. To our knowledge, the Legionella outbreak in 2005 in Norway is the largest outbreak reported until now in the Nordic countries.
